# The genome sequence of the clay, *Mythimna ferrago* (Fabricius, 1787)

**DOI:** 10.12688/wellcomeopenres.17923.1

**Published:** 2022-07-04

**Authors:** Douglas Boyes, University of Oxford and Wytham Woods Genome Acquisition Lab, Darwin Tree of Life Barcoding collective, Wellcome Sanger Institute Tree of Life programme, Wellcome Sanger Institute Scientific Operations: DNA Pipelines collective, Tree of Life Core Informatics collective, Melanie Gibbs, Darwin Tree of Life Consortium

**Affiliations:** 1 UK Centre for Ecology and Hydrology, Wallingford, Oxford, UK

**Keywords:** Mythimna ferrago, the clay, genome sequence, chromosomal, Arthropoda

## Abstract

We present a genome assembly from an individual female *Mythimna ferrago* (the clay; Arthropoda; Insecta; Lepidoptera; Noctuidae). The genome sequence is 861 megabases in span. The majority of the assembly (99.98%) is scaffolded into 32 chromosomal pseudomolecules, with the W and Z chromosomes assembled. The complete mitochondrial genome was also assembled and is 15.3 kilobases in length. Gene annotation of this assembly on Ensembl has identified 14,075 protein coding genes.

## Species taxonomy

Eukaryota; Metazoa; Ecdysozoa; Arthropoda; Hexapoda; Insecta; Pterygota; Neoptera; Endopterygota; Lepidoptera; Glossata; Ditrysia; Noctuoidea; Noctuidae; Hadeninae; Mythimna; *Mythimna ferrago* (Fabricius, 1787) (NCBI:txid997540).

## Background

The Clay, *Mythimna ferrago* (Fabricius, 1787) is a common, nocturnal, non-pest, macro-moth species that occurs across the Palearctic. In Great Britain, *M. ferrago* has been assessed against the International Union for Conservation of Nature (IUCN) Red List criteria, and categorised as a resident species of Least Concern ( Fox *et al.*, 2021). The larvae feed on grasses ( Robinson *et al.*, 2010), and overwinter as small larvae. The adult flight period is July and August. *Mythimna ferrago* can be found in a range of open habitats, including woodland, grassland, scrub, heathland, gardens and farmland.

Moths are important indicators of land-use and climate change ( Wagner *et al.*, 2021), used to study site-specific stressors such as light pollution ( Boyes *et al.*, 2021), pesticide use, and the effectiveness of agri-environment management schemes ( Botham *et al.*, 2015; Merckx *et al.*, 2009; Staley *et al.*, 2016). Within the genus *Mythimna, M. ferrago* is unusual in that, on the basis of DNA barcoding data, it forms two distinct clusters across its entire range that do not correspond with a geographical pattern ( Huemer *et al.*, 2019). Analyses at the genomic level could contribute to elucidating what drives their intra-specific variation and key genes under selection.

## Genome sequence report

**Figure 1.**
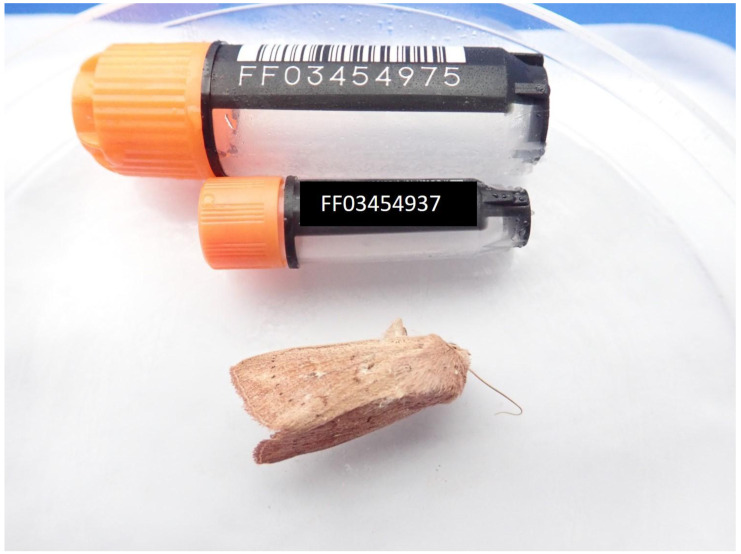
Image of the *Mythimna ferrago* specimen taken prior to preservation and processing.

The genome was sequenced from a single female *M. ferrago,* collected from Wytham Woods, Berkshire, UK ( Figure 1). A total of 31-fold coverage in Pacific Biosciences single-molecule HiFi long reads and 51-fold coverage in 10X Genomics read clouds were generated. Primary assembly contigs were scaffolded with chromosome conformation Hi-C data. Manual assembly curation corrected 15 missing/misjoins and removed 1 haplotypic duplications, reducing the assembly size by 0.99% and the scaffold number by 18.52%, and increasing the scaffold N50 by 1.95%.

**Figure 2.**
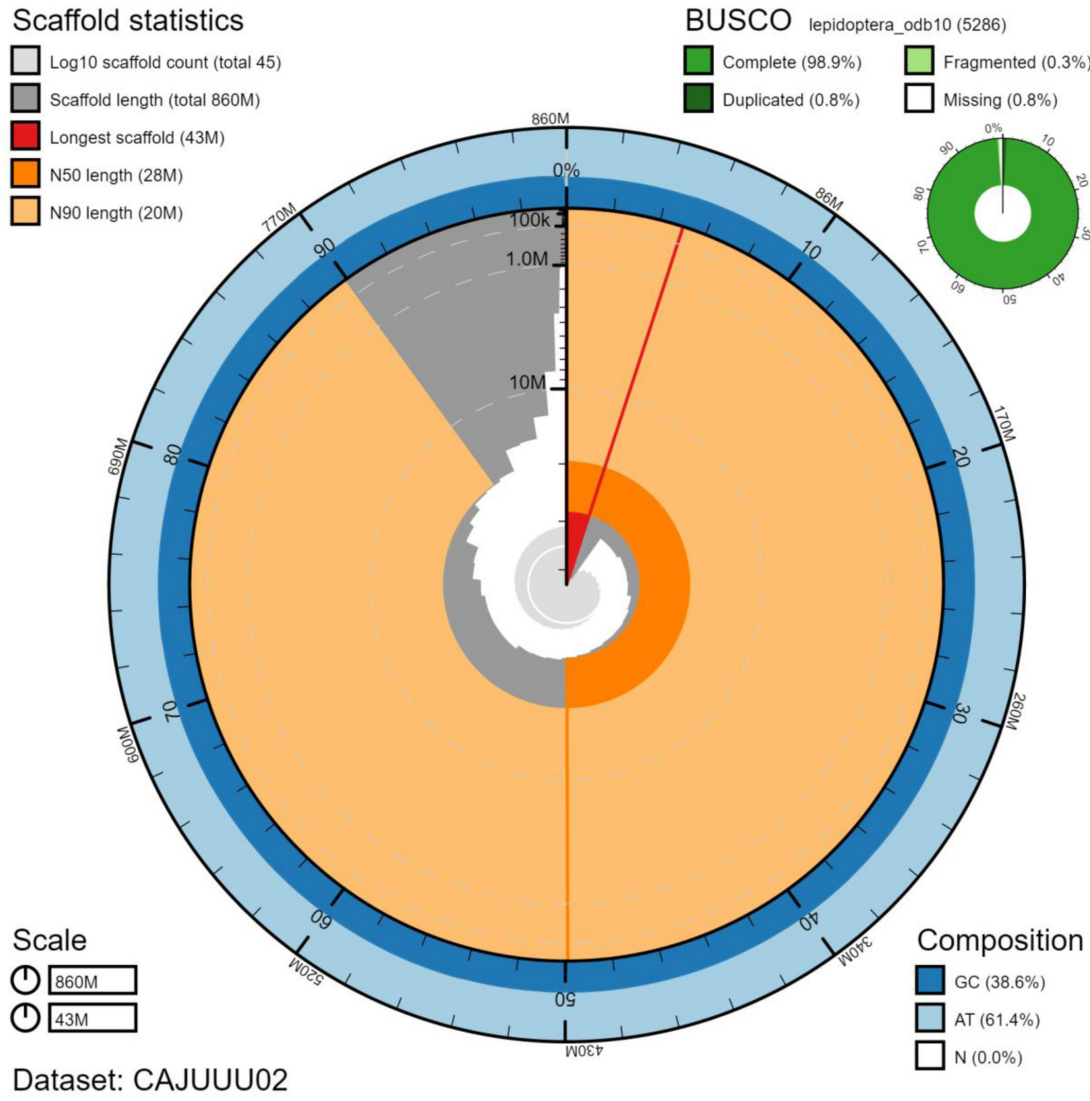
Genome assembly of *Mythimna ferrago*, ilMytFerr1.2: metrics. The BlobToolKit Snailplot shows N50 metrics and BUSCO gene completeness. The main plot is divided into 1,000 size-ordered bins around the circumference with each bin representing 0.1% of the 860,988,366 bp assembly. The distribution of chromosome lengths is shown in dark grey with the plot radius scaled to the longest chromosome present in the assembly (43,351,033 bp, shown in red). Orange and pale-orange arcs show the N50 and N90 chromosome lengths (27,874,102 and 19,573,539 bp), respectively. The pale grey spiral shows the cumulative chromosome count on a log scale with white scale lines showing successive orders of magnitude. The blue and pale-blue area around the outside of the plot shows the distribution of GC, AT and N percentages in the same bins as the inner plot. A summary of complete, fragmented, duplicated and missing BUSCO genes in the lepidoptera_odb10 set is shown in the top right. An interactive version of this figure is available at https://blobtoolkit.genomehubs.org/view/ilMytFerr1.2/dataset/CAJUUU02/snail#Filters.

The final assembly has a total length of 861 Mb in 44 sequence scaffolds with a scaffold N50 of 27.9 Mb ( Table 1). The majority, 99.98%, of the assembly sequence was assigned to 32 chromosomal-level scaffolds, representing 30 autosomes (numbered by sequence length) and the W and Z sex chromosomes ( Figure 2– Figure 5; Table 2).

**Table 1.** Genome data for *Mythimna ferrago*, ilMytFerr1.2.

*Project accession data*	
Assembly identifier	ilMytFerr1.2
Species	*Mythimna ferrago*
Specimen	ilMytFerr1 (genome assembly; Hi-C)
NCBI taxonomy ID	1789172
BioProject	PRJEB45178
BioSample ID	SAMEA7701536
Isolate information	Adult female. Abdomen (genome sequencing); head/thorax (Hi-C)
*Raw data accessions*	
PacificBiosciences SEQUEL II	ERR6436381
10X Genomics Illumina	ERR6054871-ERR6054874
Hi-C Illumina	ERR6054870
*Genome assembly*	
Assembly accession	GCA_910589285.1
*Accession of alternate haplotype*	GCA_910589535.1
Span (Mb)	861
Number of contigs	60
Contig N50 length (Mb)	27.2
Number of scaffolds	44
Scaffold N50 length (Mb)	27.9
Longest scaffold (Mb)	41.4
BUSCO * genome score	C:98.9%[S:98.1%,D:0.8%],F:0.3%,M:0.8%,n:5286
*Genome annotation*	
Number of protein-coding genes	14,075
Average length of coding sequence (bp)	20314.26
Average number of exons per transcript	6.64

*BUSCO scores based on the lepidoptera_odb10 BUSCO set using v5.1.2. C= complete [S= single copy, D=duplicated], F=fragmented, M=missing, n=number of orthologues in comparison. A full set of BUSCO scores is available at https://blobtoolkit.genomehubs.org/view/ilMytFerr1.2/dataset/CAJUUU02/busco#Filters.

**Figure 3.**
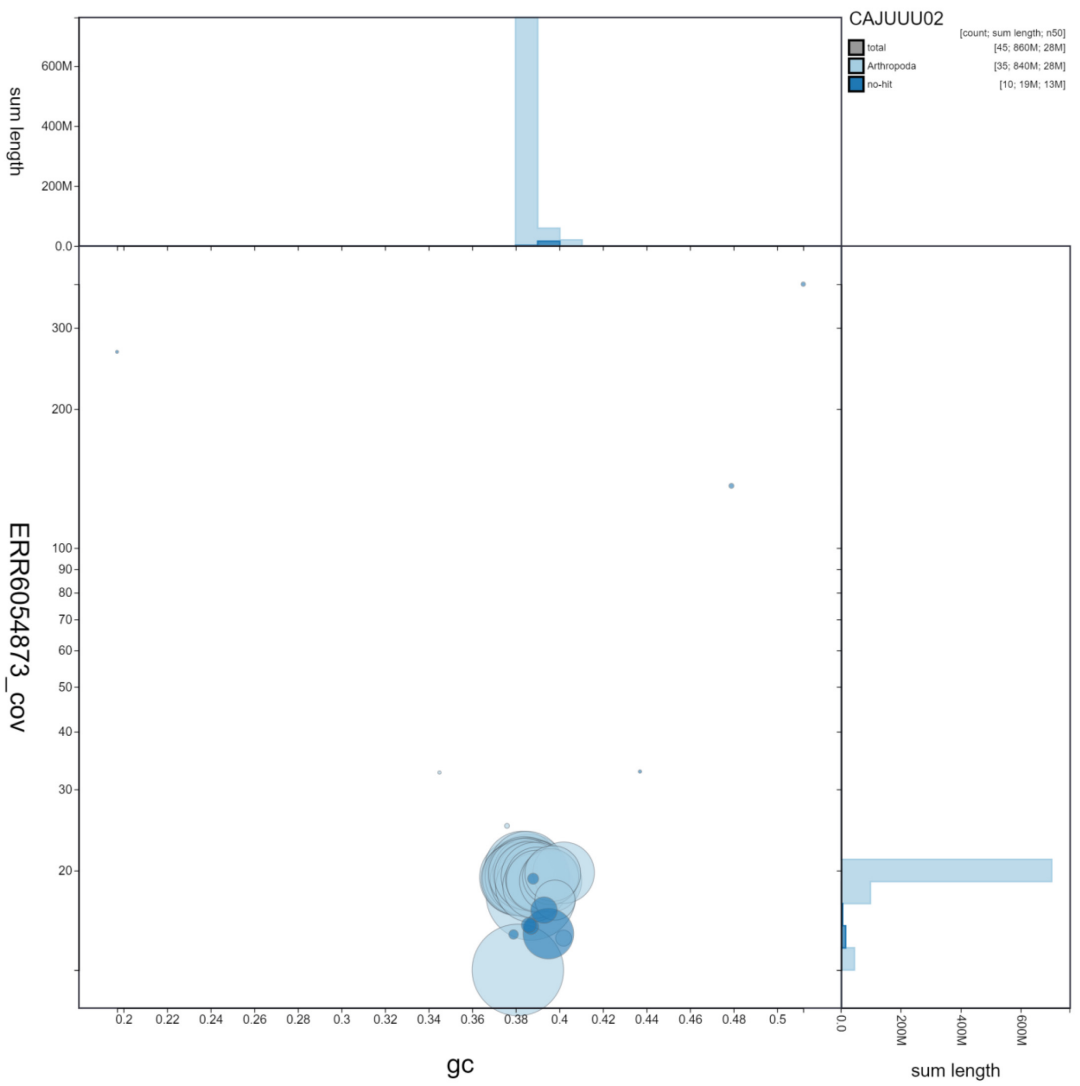
Genome assembly of *Mythimna ferrago*, ilMytFerr1.2: GC coverage. BlobToolKit GC-coverage plot. Scaffolds are coloured by phylum. Circles are sized in proportion to scaffold length. Histograms show the distribution of scaffold length sum along each axis. An interactive version of this figure is available at https://blobtoolkit.genomehubs.org/view/Mythimna%20ferrago/dataset/CAJUUU02/blob#Filters.

**Figure 4.**
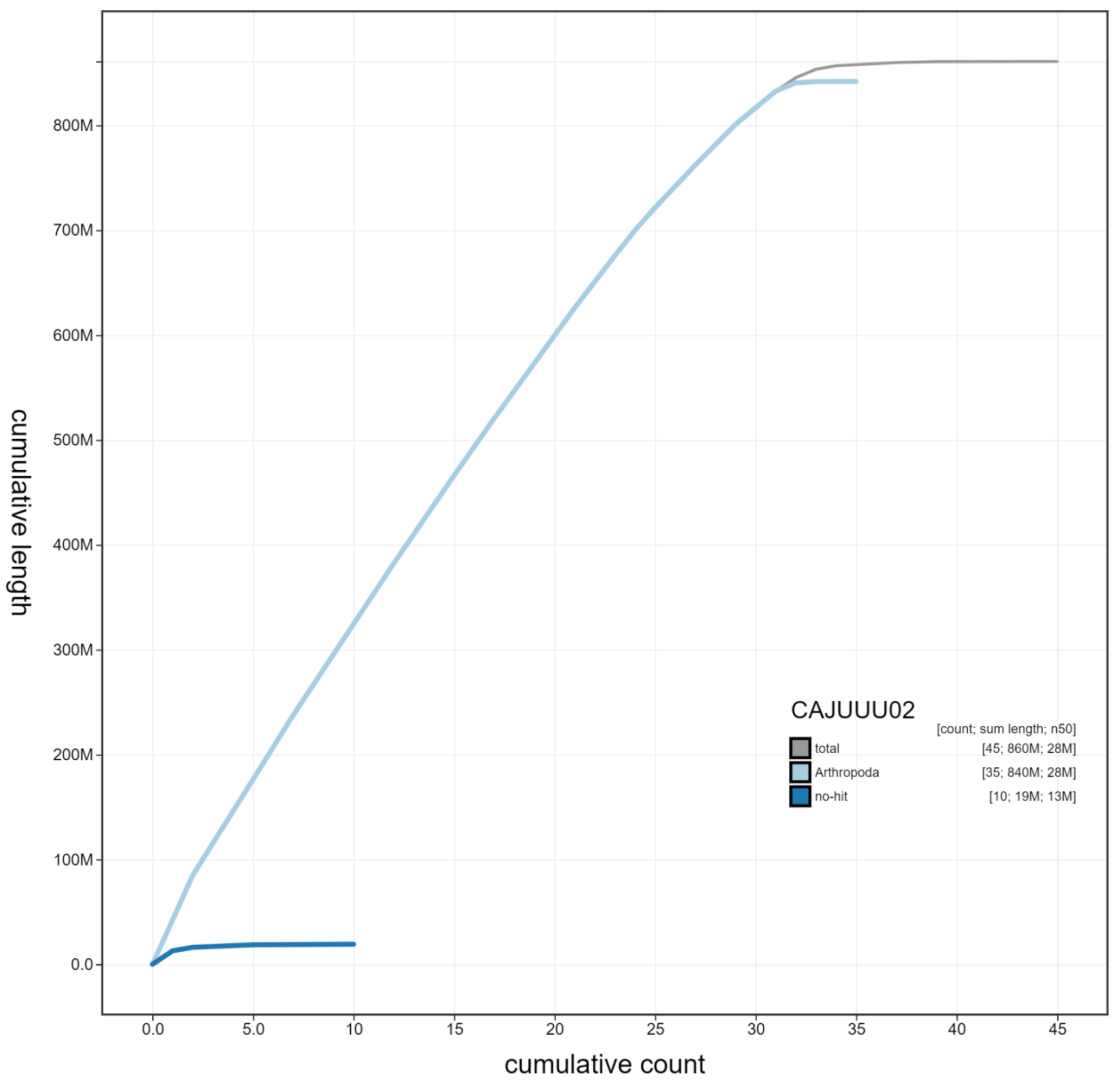
Genome assembly of *Mythimna ferrago*, ilMytFerr1.2: cumulative sequence. BlobToolKit cumulative sequence plot. The grey line shows cumulative length for all scaffolds. Coloured lines show cumulative lengths of scaffolds assigned to each phylum using the buscogenes taxrule. An interactive version of this figure is available at https://blobtoolkit.genomehubs.org/view/Mythimna%20ferrago/dataset/CAJUUU02/cumulative#Filters.

**Figure 5.**
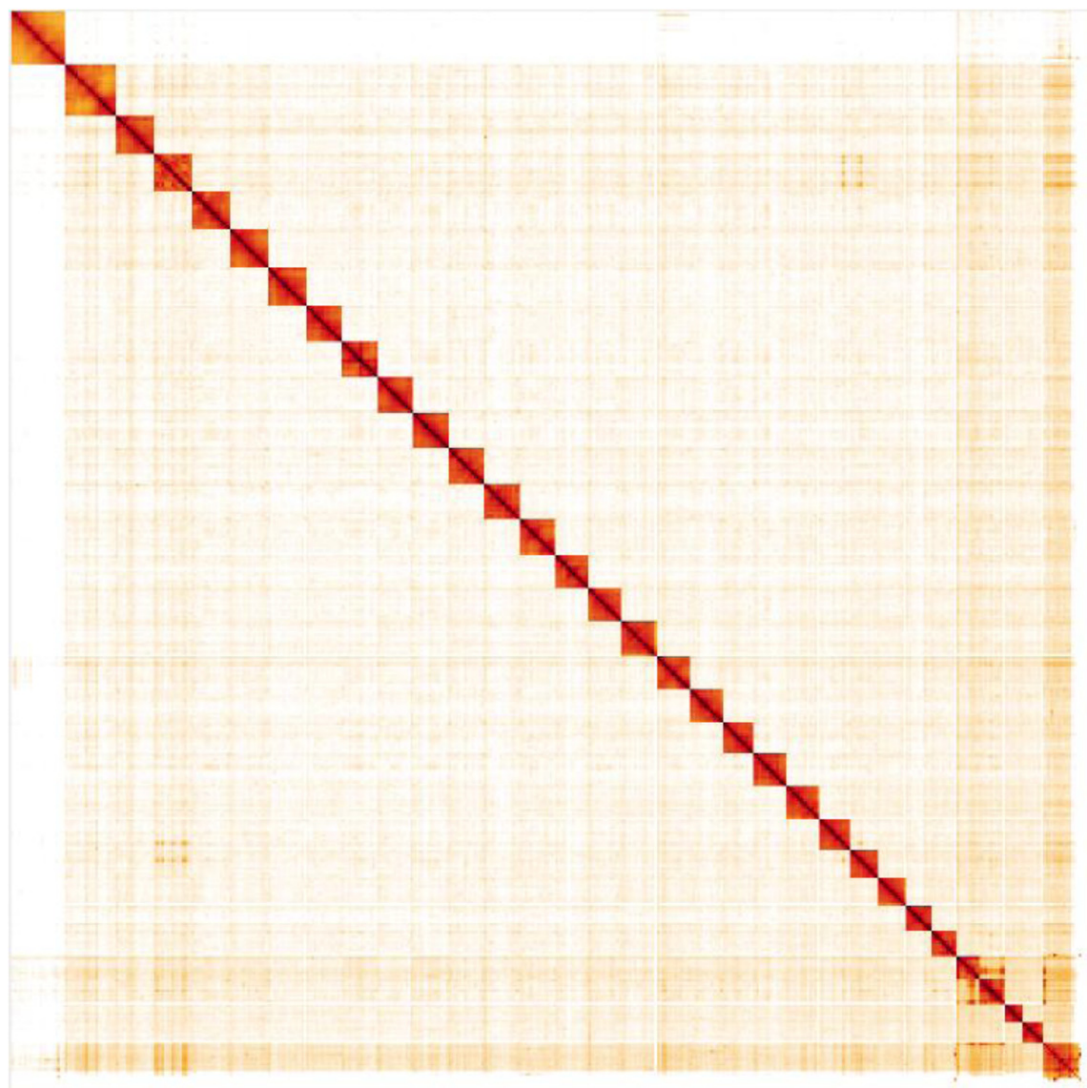
Genome assembly of *Mythimna ferrago*, ilMytFerr1.2: Hi-C contact map. Hi-C contact map of the ilMytFerr1.2 assembly, visualised in HiGlass. Chromosomes are arranged in size order from left to right and top to bottom. The interactive Hi-C map can be viewed at https://genome-note-higlass.tol.sanger.ac.uk/l/?d=UZmmjbDlR2C13gmXjFQ-xw.

**Table 2.** Chromosomal pseudomolecules in the genome assembly of *Mythimna ferrago*, ilMytFerr1.2.

INSDC accession	Chromosome	Size (Mb)	GC%
OU342674.1	1	41.45	38.7
OU342675.1	2	31.00	38.4
OU342676.1	3	30.97	38.7
OU342677.1	4	30.43	38.6
OU342678.1	5	30.31	38.5
OU342679.1	6	30.20	38.1
OU342680.1	7	29.39	38.2
OU342681.1	8	29.06	38.3
OU342682.1	9	28.89	38.2
OU342683.1	10	28.81	38.5
OU342684.1	11	28.56	38.3
OU342685.1	12	28.26	38.3
OU342686.1	13	27.87	38.4
OU342687.1	14	27.58	38.1
OU342688.1	15	27.34	38.7
OU342689.1	16	27.19	38.5
OU342690.1	17	26.42	38.8
OU342691.1	18	26.40	38.6
OU342692.1	19	26.34	38.8
OU342693.1	20	26.30	38.4
OU342694.1	21	25.67	38.7
OU342695.1	22	24.25	38.6
OU342696.1	23	23.95	38.9
OU342697.1	24	21.68	38.9
OU342698.1	25	20.82	39.0
OU342699.1	26	19.74	39.0
OU342700.1	27	19.57	39.6
OU342701.1	28	19.18	40.2
OU342702.1	29	15.96	39.6
OU342703.1	30	15.72	39.7
OU342704.1	W	12.89	39.5
OU342673.1	Z	43.35	38.1
OU342705.1	MT	0.02	19.9
-	Unplaced	15.45	39.5

The assembly has a BUSCO v5.1.2 ( Manni *et al.*, 2021) completeness of 98.9% (single 98.1%, duplicated 0.8%) using the lepidoptera_odb10 reference set (n=954). While not fully phased, the assembly deposited is of one haplotype. Contigs corresponding to the second haplotype have also been deposited.

## Genome annotation report

The ilMytFerr1.2 genome has been annotated using the Ensembl rapid annotation pipeline ( Table 1; https://rapid.ensembl.org/Mythimna_ferrago_GCA_910589285.1/Info/Index). The resulting annotation includes 25,713 transcribed mRNAs from 14,075 protein-coding and 3,455 non-coding genes.

## Methods

### Sample acquisition and nucleic acid extraction

A single adult female *M. ferrago* specimen (ilMytFerr1) was collected using a light trap from Wytham Woods, Berkshire, UK (latitude 51.772, longitude -1.338) by Douglas Boyes (University of Oxford). The specimen was identified by Douglas Boyes and snap-frozen on dry ice.

DNA was extracted at the Tree of Life laboratory, Wellcome Sanger Institute. The ilMytFerr1 sample was weighed and dissected on dry ice with tissue set aside for Hi-C sequencing. Abdomen tissue was cryogenically disrupted to a fine powder using a Covaris cryoPREP Automated Dry Pulveriser, receiving multiple impacts. Fragment size analysis of 0.01-0.5 ng of DNA was then performed using an Agilent FemtoPulse. High molecular weight (HMW) DNA was extracted using the Qiagen MagAttract HMW DNA extraction kit. Low molecular weight DNA was removed from a 200-ng aliquot of extracted DNA using 0.8X AMpure XP purification kit prior to 10X Chromium sequencing; a minimum of 50 ng DNA was submitted for 10X sequencing. HMW DNA was sheared into an average fragment size between 12-20 kb in a Megaruptor 3 system with speed setting 30. Sheared DNA was purified by solid-phase reversible immobilisation using AMPure PB beads with a 1.8X ratio of beads to sample to remove the shorter fragments and concentrate the DNA sample. The concentration of the sheared and purified DNA was assessed using a Nanodrop spectrophotometer and Qubit Fluorometer and Qubit dsDNA High Sensitivity Assay kit. Fragment size distribution was evaluated by running the sample on the FemtoPulse system.

### Sequencing

Pacific Biosciences HiFi circular consensus and 10X Genomics Chromium read cloud sequencing libraries were constructed according to the manufacturers’ instructions. Sequencing was performed by the Scientific Operations core at the Wellcome Sanger Institute on Pacific Biosciences SEQUEL II (HiFi) and Illumina NovaSeq 6000 (10X) instruments. Hi-C data were generated in the Tree of Life laboratory from head/thorax tissue of ilMytFerr1 using the Arima v2 kit and sequenced on a NovaSeq 6000 instrument.

### Genome assembly

Assembly was carried out with Hifiasm ( Cheng *et al.*, 2021); haplotypic duplication was identified and removed with purge_dups ( Guan *et al.*, 2020). One round of polishing was performed by aligning 10X Genomics read data to the assembly with longranger align, calling variants with freebayes ( Garrison & Marth, 2012). The assembly was then scaffolded with Hi-C data ( Rao *et al.*, 2014) using SALSA2 ( Ghurye *et al.*, 2019). The assembly was checked for contamination and corrected using the gEVAL system ( Chow *et al.*, 2016) as described previously ( Howe *et al.*, 2021). Manual curation ( Howe *et al.*, 2021) was performed using gEVAL, HiGlass ( Kerpedjiev *et al.*, 2018) and Pretext. The mitochondrial genome was assembled using MitoHiFi ( Uliano-Silva *et al.*, 2021), which performs annotation using MitoFinder ( Allio *et al.*, 2020). The genome was analysed and BUSCO scores generated within the BlobToolKit environment ( Challis *et al.*, 2020). Table 3 contains a list of all software tool versions used, where appropriate.

**Table 3.** Software tools used.

Software tool	Version	Source
Hifiasm	0.15.3	Cheng *et al.*, 2021
purge_dups	1.2.3	Guan *et al.*, 2020
SALSA2	2.2	Ghurye *et al.*, 2019
longranger align	2.2.2	https://support.10xgenomics.com/genome-exome/ software/pipelines/latest/advanced/other-pipelines
freebayes	1.3.1-17-gaa2ace8	Garrison & Marth, 2012
MitoHiFi	2.0	Uliano-Silva *et al.*, 2021
HiGlass	1.11.6	Kerpedjiev *et al.*, 2018
PretextView	0.2.x	https://github.com/wtsi-hpag/PretextView
BlobToolKit	3.0.5	Challis *et al.*, 2020

### Genome annotation

The Ensembl gene annotation system ( Aken *et al.*, 2016) was used to generate annotation for the *Mythimna Ferrago* assembly (GCA_910589285.1). Annotation was created primarily through alignment of transcriptomic data to the genome, with gap filling via protein-to-genome alignments of a select set of proteins from UniProt ( UniProt Consortium, 2019).

### Ethics/compliance issues

The materials that have contributed to this genome note have been supplied by a Darwin Tree of Life Partner. The submission of materials by a Darwin Tree of Life Partner is subject to the Darwin Tree of Life Project Sampling Code of Practice. By agreeing with and signing up to the Sampling Code of Practice, the Darwin Tree of Life Partner agrees they will meet the legal and ethical requirements and standards set out within this document in respect of all samples acquired for, and supplied to, the Darwin Tree of Life Project. Each transfer of samples is further undertaken according to a Research Collaboration Agreement or Material Transfer Agreement entered into by the Darwin Tree of Life Partner, Genome Research Limited (operating as the Wellcome Sanger Institute), and in some circumstances other Darwin Tree of Life collaborators.

## Data availability

European Nucleotide Archive: Mythimna ferrago (the clay). Accession number PRJEB45178; https://identifiers.org/ena.embl/PRJEB45178.

The genome sequence is released openly for reuse. The *M. ferrago* genome sequencing initiative is part of the Darwin Tree of Life (DToL) project. All raw sequence data and the assembly have been deposited in INSDC databases. Raw data and assembly accession identifiers are reported in Table 1.

## Author information

Members of the University of Oxford and Wytham Woods Genome Acquisition Lab are listed here : https://doi.org/10.5281/zenodo.6418202.

Members of the Darwin Tree of Life Barcoding collective are listed here: https://doi.org/10.5281/zenodo.6418156.

Members of the Wellcome Sanger Institute Tree of Life programme are listed here: https://doi.org/10.5281/zenodo.6418327.

Members of Wellcome Sanger Institute Scientific Operations: DNA Pipelines collective are listed here: https://doi.org/10.5281/zenodo.5746904.

Members of the Tree of Life Core Informatics collective are listed here: https://doi.org/10.5281/zenodo.6125046.

Members of the Darwin Tree of Life Consortium are listed here: https://doi.org/10.5281/zenodo.6418363.
