# Supplementary material for: The genome sequence of the clay, Mythimna ferrago (Fabricius, 1787)
Source: Wellcome Open Res. 2022 Jul 4;7:177. [Version 1] doi: 10.12688/wellcomeopenres.17923.1 (PMC9382153; doi:10.12688/wellcomeopenres.17923.1)
Supplement: Peer review history

# Peer review: Reviewer response for version 1

**DOI:** 10.21956/wellcomeopenres.19861.r51418

**Published:** 2022-08-16

**Authors:** Niclas Backström

**Affiliations:** 1 Department of Evolutionary Biology, Evolutionary Biology Centre (EBC), Uppsala University, Uppsala, Sweden

The article describes the statistics of a genome assembly of the clay (Mythimna ferrago) and is written in standard format for the DToL genome notes. The paper is well written and the results are clearly presented. Accession information for all data sets is also available in the note. The quality of the assembly is impressive and the data will definitely be useful for forthcoming intra-specific population genomic studies and for comparative genomics studies in Lepidoptera in general.

 Minor comments:

 1) It's a bit unclear to me what average length of coding sequence refers to (Table 1). Is this the average length of the entire gene (introns and UTR included) or the sum of lengths of all coding exons (probably not since it is > 20 kb), or something else? Maybe a short note in the table legend can be added to make this clear.

 2) Methods: Section 1, first sentence. "using a light trap from Wytham Woods" sounds a bit like the light trap was from WW and not that the sample site was WW?

 3) Methods: Genome annotation paragraph. Unclear if the transcriptomic data are from the same species?

 4) Methods: Genome annotation paragraph. Maybe specify which set of proteins from UniProt that was used for the alignments?

Are sufficient details of methods and materials provided to allow replication by others?

Yes

Is the rationale for creating the dataset(s) clearly described?

Yes

Are the datasets clearly presented in a useable and accessible format?

Yes

Are the protocols appropriate and is the work technically sound?

Yes

Reviewer Expertise:

Evolutionary genomics, population genetics, molecular evolution

I confirm that I have read this submission and believe that I have an appropriate level of expertise to confirm that it is of an acceptable scientific standard.

# Peer review: Reviewer response for version 1

**DOI:** 10.21956/wellcomeopenres.19861.r51417

**Published:** 2022-07-08

**Authors:** Steven M. Van Belleghem

**Affiliations:** 1 Department of Biology, Rio Piedras Campus, University of Puerto Rico, San Juan, Puerto Rico; 2 Department of Biology, KU Leuven, Leuven, Belgium

The note reports on the the assembly of the genome of the clay butterfly through the initiative of the Darwin Tree of Life Barcoding collective. The assembly included PacBio Hifi, 10X and Hi-C data and resulted in a high quality assembly that was further manually curated to remove minor assembly mistakes.

 Comments:

 Please clarify how the W and Z chromosome were identified.

Are sufficient details of methods and materials provided to allow replication by others?

Yes

Is the rationale for creating the dataset(s) clearly described?

Yes

Are the datasets clearly presented in a useable and accessible format?

Yes

Are the protocols appropriate and is the work technically sound?

Yes

Reviewer Expertise:

Genomics, adaptation

I confirm that I have read this submission and believe that I have an appropriate level of expertise to confirm that it is of an acceptable scientific standard.
